# Influences on single-use and reusable cup use: a multidisciplinary mixed-methods approach to designing interventions reducing plastic waste

**DOI:** 10.14324/111.444/ucloe.000025

**Published:** 2021-09-22

**Authors:** Ayşe Lisa Allison, Fabiana Lorencatto, Mark Miodownik, Susan Michie

**Affiliations:** 1 UCL Plastic Waste Innovation Hub, University College London, London, UK; 2 UCL Centre for Behaviour Change, University College London, London, UK; 3 UCL Mechanical Engineering, University College London, London, UK

**Keywords:** single-use, reusable, coffee cups, plastic waste, circular economy, intervention, behaviour change, influences, COM-B, Behaviour Change Wheel

## Abstract

An estimated 2.5–5 billion single-use coffee cups are disposed of annually in the UK, most of which consist of paper with a plastic lining. Due to the difficulty of recycling poly-coated material, most of these cups end up incinerated or put in landfills. As drinking (take-away) hot beverages is a behaviour, behaviour change interventions are necessary to reduce the environmental impacts of single-use coffee cup waste. Basing the design of interventions on a theoretical understanding of behaviour increases the transparency of the development process, the likelihood that the desired changes in behaviour will occur and the potential to synthesise findings across studies. The present paper presents a methodology for identifying influences on using single-use and reusable cups as a basis for designing intervention strategies. Two behaviour change frameworks: The Theoretical Domains Framework (TDF) and the Capability-Opportunity-Motivation-Behaviour (COMB) model of behaviour, were used to develop an online survey and follow-up interviews. Research findings can inform the selection of intervention strategies using a third framework, the Behaviour Change Wheel (BCW). The application of the methodology is illustrated in relation to understanding barriers and enablers to single-use and reusable cup use across the setting of a London university campus. We have developed a detailed method for identifying behavioural influences relevant to pro-environmental behaviours, together with practical guidance for each step and a worked example. Benefits of this work include it providing guidance on developing study materials and collecting and analysing data. We offer this methodology to the intervention development and implementation community to assist in the application of behaviour change theory to interventions.

## Introduction

Tea and coffee consumption in the UK have become increasingly ‘on the go’ [1]. This has led to a rise in the number of hot drinks sold in cups intended for single use – an estimated 2.5–5 billion single-use coffee cups are disposed of annually in the UK, most consisting of a paper body and plastic lining [2]. Recycling these cups, although technically possible, is limited by a lack of facilities in the UK capable of separating the materials for recycling [2]. Automatic sorting and collecting also pose a challenge [3,4]. The lack of infrastructure to cope with this type of waste means that most single-use cups end up littered, incinerated or in landfill, contributing to environmental degradation [5]. In addition, the carbon dioxide emissions generated by single-use coffee cups are approximately 1.5 times the weight of the cup [6]. Reducing the number of single-use cups in circulation is therefore important for reaching net zero targets [7]. As using single-use cups is a behaviour, behaviour change interventions are necessary to reduce the environmental impacts of single-use coffee cup waste.

There are some preliminary published examples of interventions aimed at reducing use of single-use coffee cups within the scientific literature. These have focussed on the promotion of reusable alternatives. Examples include interventions promoting use of reusable cups across a university campus in Wales [8] and Australia [9]. While these interventions efforts provide useful insights, the results may not be transferable to other university contexts, and they were not designed on a comprehensive understanding of the various barriers and facilitators to using reusable cups within their given university contexts. Behaviour change interventions do not occur in a social vacuum [10]. Aside from differing socio-cultural contexts, the physical environmental contexts of interventions aimed at changing cup use can vary greatly across more tightly knit ‘closed loop’ campus environments versus a university where the campus is spread across a busy metropolis. For instance, in the latter, university catering outlets may be littered amongst other cafes and catering outlets, creating additional challenges to implementation. For example, a single-use coffee cup surcharge implemented in city university cafes could have the unintended consequence of shifting people towards purchasing their hot drinks at other, non-university, catering outlets where such a charge does not exist. In more ‘closed loop’ environments, this extraneous factor may be easier to control for due to a lack of alternatives.

In addition, building an intervention on a theory and evidence informed understanding of behaviour may increase the potential of such interventions being more effective. Aside from the physical context of the intervention, this seemingly simple behaviour of using a reusable cup is located within a complex system of several interacting groups of actors operating at various organisational levels. Guidance for developing and evaluating the kinds of ‘complex’ interventions needed to tackle this type of system point to the importance of grounding interventions in both theory and evidence, local and more general [11,12]. Progress in this area is therefore likely to benefit from formative research to develop understanding of the factors influencing this behaviour in its given context. This way, it is possible to develop interventions that are targeted at the appropriate individual, socio-cultural and contextual influences on a given behaviour.

The purpose of this paper is to present a methodology which can provide the underpinning evidence for a theory of the factors influencing single-use and reusable cup use. By starting from a more comprehensive understanding of the factors influencing a behaviour in its given context, it is more likely that interventions will be effective at changing behaviour.

To this end, our aims are to present a methodology that identifies:

- Current behaviour with respect to single-use and reusable cup use;
- The various capability, opportunity and motivation related influences on single-use and reusable cup use;
- People’s views on potential intervention strategies to promote reusable cup use.

### Literature review

**Figure 1:**
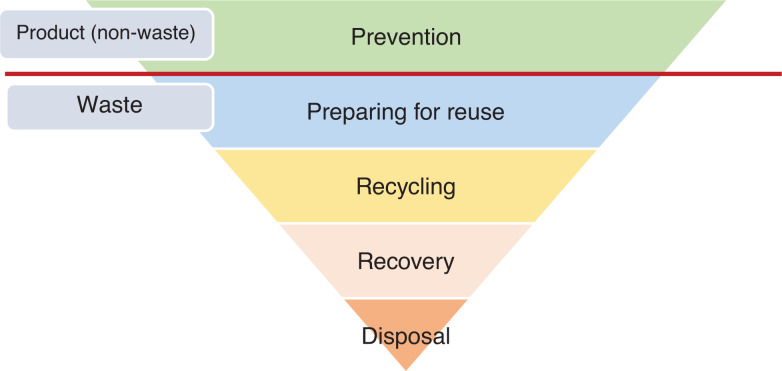
The Waste Hierarchy as set out in Article 4 of the revised Waste Framework (Directive 2008/98/EC).

Preventative waste management approaches have been identified as more effective and economical than strategies aimed at recovering materials, in particular when they are high volume and low value [13]. For instance, the ‘waste hierarchy’ set out in Article 4 of the European Union’s (EU’s) revised Waste Framework (Directive 2008/98/EC) [14], which ranks waste management options according to what is best for the environment (shown in Fig. 1), identifies item re-use as the optimal strategy to reduce waste once a product has entered circulation. This hierarchy recommends waste management strategies that prioritise reducing the amount of waste in circulation, rather than managing it once it is there. When waste is created, the Waste Hierarchy gives priority to preparing it for re-use, then recycling, then recovery and last of all disposal (e.g., landfill, incineration).

Citizen behaviour change with respects to ‘on-the-go’ hot beverage consumption (i.e., switching from single-use to reusable) therefore plays a key role in reducing the amount of waste from single-use cups. Life cycle assessments have shown the environmental impacts of different types of cups to vary depending on the impact categories investigated [15]. Examples of different impact categories include stratospheric ozone depletion, resource consumption (e.g., land and water use), ecotoxicity and waste [16]. Evidence suggests that replacing single-use plastic cups for reusable ones can significantly reduce waste generation (though this may increase water consumption) [17]. As highlighted above, citizen behaviour change will be key to transition from using single-use cups to using reusable cups. To effectively change behaviour (i.e., design an intervention) we first need to understand why behaviour is as it is and what it would take to bring about the desired change. Using suitable behaviour change intervention development frameworks can aid the process of identifying behavioural influences that need to be targeted for change to occur.

**Figure 2:**
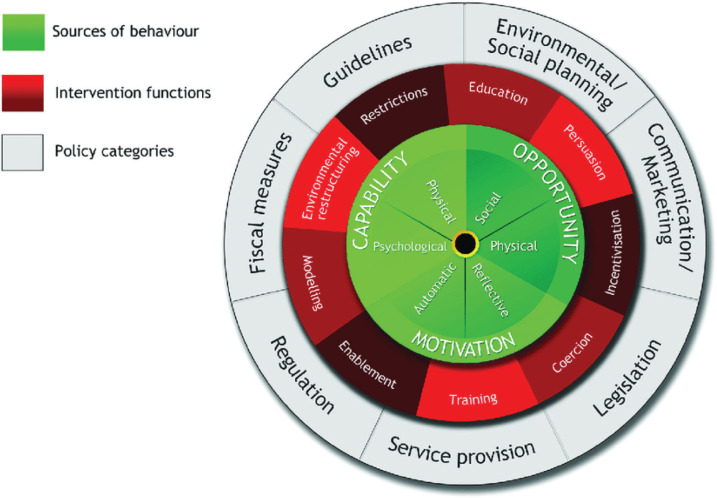
The BCW – a framework for intervention development, evaluation and evidence synthesis [10,18].

Shown in Fig. 2, the Behaviour Change Wheel (BCW) is an integrated synthesis of 19 other behavioural frameworks. It provides a structured approach for conceptualising problems in behavioural terms and designing behaviour change interventions for individuals, organisations and populations. The wheel itself consists of three parts: 1) An inner hub, which represents, in terms of capability, opportunity and motivation, what needs to be targeted to achieve the desired behaviour change; 2) A middle layer of intervention types, which are broad categories of approach to changing these targets; and 3) An outer layer, which are policy options for leveraging these broad types of intervention.

In terms of method, the BCW advocates three key steps: 1) Behavioural target specification: Identify the precise target(s) of the intervention in terms of what behaviour(s) need(s) to change, to what degree, in what way, in whom and for how long. 2) Behavioural diagnosis: Finding out what would need to change for the behaviour to change in terms of the Capability, Opportunity, Motivation, Behaviour model (COM-B). 3) Intervention development: Using the behavioural diagnosis to select intervention types, policy categories and component behaviour change techniques (elementary components of interventions such as goalsetting, providing rewards, etc.) from the Behaviour Change Techniques Taxonomy [3].

**Figure 3:**
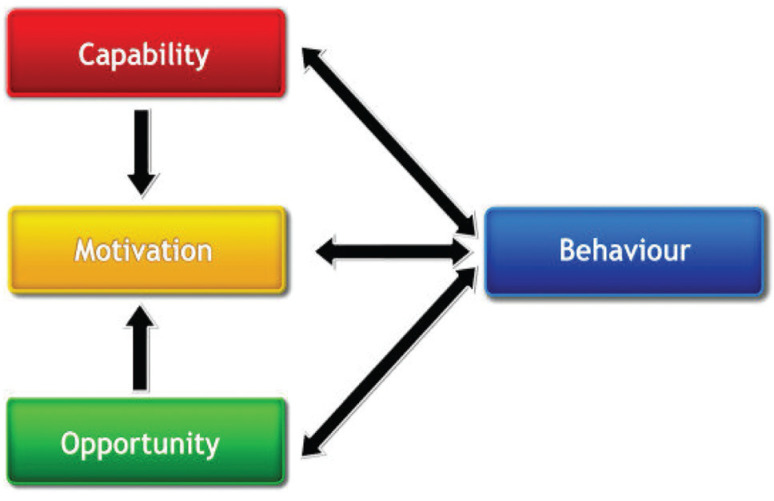
The COM-B model – a framework for understanding behaviour [10,18].

As represented in the inner hub of the BCW, the COM-B model [10,18] was developed as part of this wider intervention development process (shown in Fig. 3). The COM-B model provides a useful framework for identifying the various individual, socio-cultural and situational influences on a behaviour and can be used to identify behavioural targets for interventions. The model posits that for a behaviour to occur, there must be: Capability, Opportunity and Motivation to enact the behaviour. Capability can refer to people’s physical or psychological capability such as their physique and stamina or knowledge, intellectual capacity and memory and decision-making processes. Opportunity can refer to social or physical opportunity such as the social environment of cultures and norms or the physical environment of objects and events with which people interact. Motivation can be automatic or reflective motivation and refers to the intentions, desires, evaluations, habits and instincts that direct human behaviour.

These COM-B categories can be elaborated into the Theoretical Domains Framework (TDF) [19], shown in Table 1. It includes 14 Theoretical Domains, representing individual, socio-cultural and environmental factors influencing behaviour. These include people’s knowledge and skills, memory, attention and decision-making processes, beliefs about capabilities and consequences, goals and emotions as well as physical and social environmental factors.

**Table 1.** The Theoretical Domains Framework – 14 domains of individual, socio-cultural and environmental influences on a behaviour [19]

TDF domain	Explanation
Knowledge	An awareness of the existence of something
Skills	An ability or proficiency acquired through practice
Social/professional role and identity	A coherent set of behaviours and displayed personal qualities of an individual in a social or work setting
Beliefs about capabilities	Acceptance of the truth, reality or validity about an ability, talent or facility that a person can put to constructive use
Optimism	The confidence that things will happen for the best or that desired goals will be attained
Beliefs about consequences	Acceptance of the truth, reality or validity about outcomes of a behaviour in a given situation
Reinforcement	Increasing the probability of a response by arranging a dependent relationship, or contingency, between the response and a given stimulus
Intentions	A conscious decision to perform a behaviour or a resolve to act in a certain way
Goals	Mental representations of outcomes or end states that an individual wants to achieve
Memory, attention and decision processes	The ability to retain information, focus selectively on aspects of the environment and choose between two or more alternatives
Environmental context and resources	Any circumstance of a person’s situation or environment that discourages or encourages the development of skills and abilities, independence, social competence and adaptive behaviour
Social influences	Those interpersonal processes that can cause individuals to change their thoughts, feelings, or behaviours
Emotion	A complex reaction pattern, involving experiential, behavioural, and physiological elements, by which the individual attempts to deal with a personally significant matter or event
Behavioural regulation	Anything aimed at managing or changing objectively observed or measured actions

**Figure 4:**
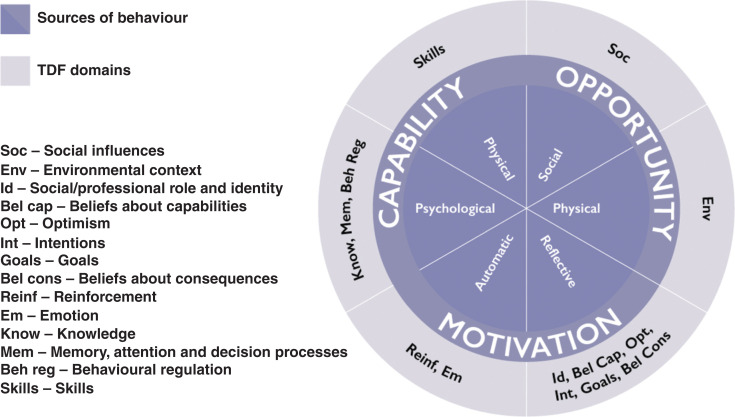
Fourteen TDF domains linked to COM-B categories.

The relationship between COM-B categories and TDF domains are shown in Fig. 4. COM-B and TDF may be considered as part of the ‘toolbox’ of behavioural science frameworks that can be used to conduct a ‘behavioural diagnosis’ (i.e., understand the influences on behaviour in its context) [18,20]. In the present study, we aim to use COM-B and TDF as data collection and data analysis frameworks. Research findings can inform selection of intervention strategies by using the BCW. In sharing our paper, we hope to provide an adaptable theory- and evidence-based template that can be used by other intervention practitioners and researchers.

## Method and application

### Design

We propose a mixed-methods study [21] including an online survey followed by semi-structured interviews conducted with a sample of survey respondents. Mixed methods have been defined as ‘*research in which the investigator collects and analyses data, integrates the findings and draws inferences using both qualitative and quantitative approaches or methods in a single study’* [22]. There are various reasons why researchers may opt for mixed-methods. In line with prior rationales for adopting mixed methods [23–25], we chose mixed-methods in order to achieve ‘triangulation’ (i.e., seeking corroboration between quantitative and qualitative data to increase validity of findings) and ‘completeness’ (i.e., combining research approaches to provide a more comprehensive picture of the study phenomenon). This study involves following up a quantitative phase by a qualitative phase in order to explain and explore in more detail the mechanism behind the quantitative survey results [26].

### Method

#### Phase 1: Online Survey

*Survey development.* A survey was developed in line with Atkin et al.’s guidance for using TDF in implementation research [27]. Three sources were used to develop initial survey items: a prior survey on attitudes towards reusable cups developed by our collaborators at Sheffield University; an evidence review of perceptions, behaviours and interventions related to reducing plastic waste [28]; and discussions with UCL’s Sustainability Team to understand what information would be useful to them in planning the intervention. The first section includes questions about participant demographic information and current behaviour relating to single-use reusable cups. The subsequent two sections include: open-ended questions and, statements regarding behavioural influences and possible intervention strategies to promote reusable cup use, with agreement expressed on a 5-point Likert scale.

A preliminary set of survey items were subsequently cross referenced with COM-B and TDF to ensure no likely categories of influence were being omitted from the survey. The number of TDF domains and COM-B categories covered and number of questions per domain in a data collection instrument can vary depending on the target behaviour and existing evidence [27]. For example, where prior research or key stakeholder consensus has established that a certain TDF domain/COM-B category is unlikely to be influential on a target behaviour, researchers may consider excluding questions relating to that TDF domain/COM-B category and focusing more on TDF domains/COM-B categories considered more relevant. For instance, questions relating to physical skills are unlikely to be relevant for cup use amongst a general university population. As such, we omitted questions relating to physical capability (COM-B), ensuring the survey was as short as possible in order to encourage a higher completion rate and as well as more thoughtful responses for the included items [29]. To counter this potential limitation, we included an open-ended question where participants could mention factors influencing their behaviour that may not have been covered by our survey. Table 2 shows the relationship between our survey items, psychological constructs, COM-B categories and TDF domains. The final version of the survey is openly available via Open Science Framework (OSF) at https://osf.io/ujkwe/.

**Table 2.** Survey items, related constructs, TDF domains and COM-B categories

COM-B category	TDF domain	Construct	Survey item	Rationale
Psychological capability	Knowledge	n/a	n/a	n/a
	Memory, attention, decision processes	Memory [1]	• I’m likely to forget to take a reusable cup with me	Adapted from O’Brian et al. [30], Cane et al. [19] and Michie et al. [18]
	Behavioural regulation	n/a	n/a	n/a
Physical capability	Skills	n/a	n/a	n/a
Physical opportunity	Environmental context and resources	Resources/material [4]	• I don’t have enough space to carry a reusable cup around with me all day • Cleaning a reusable cup is too inconvenient • Reusable cups are too expensive to buy • There aren’t enough facilities on campus to clean reusable cups	Adapted from Oliveira et al. [31] Cane et al. [19] and Michie et al. [18]
Social opportunity	Social influences	Descriptive norms [1]	• Most of my colleagues/friends don’t use a reusable cup	Adapted from Wakefield et al. [32] and Cialdini et al. [33]
Automatic motivation	Emotion	Affect [1]	• I feel guilty if I buy a drink in a single-use cup	Adapted from Wakefield et al. [32] and Russell et al. [34]
	Reinforcement	Reinforcement [1]	• I don’t like how my drink tastes in a reusable cup	Adapted from Skinner et al. [35] Wakefield et al. [32]
Reflective motivation	Social/professional role and identity	Role/identity [1]	• I feel good about myself when I use a reusable cup	Adapted from Cane et al. [19] and Wakefield et al. [32]
	Beliefs about capabilities	n/a	n/a	n/a
	Beliefs about consequences	Attitudes [4]	• I think reusable cups are good for the environment • I think single-use cups are harmful for the environment • Reusable cups don’t look as good as single use cups • I don’t think reusable cups to be hygienic	Adapted from Wakefield et al. [32] Ajzen et al. [36]
	Optimism	Outcome expectancies [2]	• It makes no difference to the environment whether I use a reusable cup or not • A reusable cup may leak in my bag	Adapted from Bandura et al. [37] and Wakefield et al. [32]
	Intention	Intention [1]	• ‘Would you like to own a reusable cup?’ [Definitely yes/Probably yes/Not sure/Probably not/Definitely not]	Adapted from Cane et al. [19] and Ajzen et al. [36]
	Goals	Priorities [1]	• I have too many other things to think about other than the type of cup I buy my hot drinks in	Adapted from West et al. [38] and Wakefield et al. [32]

A hardcopy of the survey was piloted for comprehensibility and feasibility with a sample of UCL students and staff including members of the UCL Plastic Waste Innovation Hub and UCL Sustainability. A digital version, built on Qualtrics [39], was piloted for usability with the same sample of students and staff and a group of behaviour change experts.

*Participants.* Convenience sampling [40] will be used to recruit participants for the survey. Participants will include university students and staff. Exclusion criteria include being under 18 years of age, having completed the survey previously and not having sufficient English to complete the survey. Entering into a prize draw for gift vouchers will be used as an incentive for survey completion.

We will aim for a minimum total sample size of 172 survey respondents. This is based on a G*Power [41] sample size calculation for a fixed model multiple linear regression with the parameters of effect size = 0.15 (medium), a = 0.05, power = 0.95, number of predictors = 10. These parameters were chosen in line with prior guidance for choosing effect size, power and significance parameters in sample size calculations [42]. We chose 10 predictors, for each of the 10 psychological constructs being measured in Table 2.

*Procedure.* We will advertise the study using UCL social media and email. An advert containing a link to the survey will be posted in a select number of undergraduate and postgraduate Facebook groups and advertised via UCL Twitter pages. In addition, invitation emails containing the survey link will be circulated to a select number of students and staff drawn from a select number of university mailing lists. Informed consent will be obtained from all participants prior to data collection. After completion, participants will be asked to leave their university email addresses if they were willing to be contacted about follow-up interviews and take part in the prize draw.

*Analysis.* To identify current behaviour with respect to single-use and reusable cup use, responses will be summarised using frequencies and percentages.

To identify the various capability-, opportunity- and motivation-related influences on single-use and reusable cup use, we will compute the mean scale scores for each COM-B category and conduct exploratory factor analyses to assess the internal consistency of survey items. Responses across participant groups, for example, staff versus students will be compared. To identify COM-B categories associated with cup use, we will conduct fixed model multiple linear regression analyses with COM-B categories and psychological constructs as the independent variables and cup use behaviour as the dependant variable. We will analyse responses to the open-ended questions via thematic analysis in line with Braun and Clarke’s guidance [43]. Any additional behavioural influences generated will be summarised as frequencies and mapped onto COM-B categories of capability, opportunity and motivation.

To identify people’s views on potential intervention strategies to promote reusable cup use we will descriptively summarise the extent to which respondents support certain intervention strategies. Open-ended responses will be analysed by categorising participants’ suggested intervention strategies according to BCW intervention types and component Behaviour Change Techniques from the Behaviour Change Techniques Taxonomy [44].

#### Phase 2: Follow-up interviews

*Participants.* Purposive sampling [40] will be used to recruit participants. From the survey respondents willing to be contacted for follow-up interviews, we will purposefully invite 15–20 participants to ensure an equal gender split across staff, undergraduates and postgraduates.

*Interview schedule development.* An interview schedule will be developed in line with guidance from Atkins et al. [27]. The interviews will explore in more depth the influences on single-use and reusable cup use identified in the survey. It will be developed based on TDF domains. It will include at least one open-ended question per domain, followed by a series of follow-up prompts. A draft topic guide is openly available via OSF showing how each of the questions are linked to TDF domains: https://osf.io/ujkwe/. Final questions will be refined, depending on the results of the survey, in order to explore the most relevant barriers and enablers to single-use and reusable cup use. We will pilot the final version of the interview guide with three students and three staff members prior to data collection.

*Procedure.* Participants will be invited for an interview and consent sought prior to the interview via their UCL emails. We will conduct interviews over an online video-conferencing platform offering end-to-end encryption, lasting an estimated 20–45 minutes. They will be audiotaped and transcribed verbatim for analysis.

*Analysis.* We will conduct an inductive thematic analysis in line with Braun and Clarke’s approach [43] and map emergent themes onto COM-B categories. Additional guidance on conducting thematic analysis can be found elsewhere [45,46]. In line with the analysis taken by others investigating influences on behaviours related to reducing plastic waste [47], below is a summary of the steps we will take:

- *Familiarisation with the data*. This involves breaking the transcript down into units of ‘utterances’, reading through all the utterances and noting down any recurring patterns;
- *Generation of initial codes to indicate themes*. As utterances are assigned codes, a coding framework detailing code labels and definitions can be developed and revised iteratively to help guide subsequent coding;
- *Searching for themes.* This involves organising codes into a tentative set of candidate themes;
- Review of themes. This involves a back-and-forth process of revisiting the raw interview data and coding framework in order to update the names, descriptions and definitions of candidate themes;
- *Mapping of emergent themes onto the COM-B categories of barriers and enablers.* In this step themes are mapped depending on whether they refer to capability, opportunity and motivation. They are barriers if they hinder the target behaviour and an enabler if they promote the target behaviour;
- *Assignation of names and definitions for themes*. This involves finalising the name, definition, description and example quotes for each theme;
- *Production of the report*. This involves writing up the analysis with feedback from <softenter>co-investigators.

### Case study description

The study setting is the central Bloomsbury campus of University College London whose sustainability strategy is to be single-use plastic free by 2024. Efforts to increase reusable cup use across the UCL campus have had varied success. First, UCL freely distributed reusable cups to students during their ‘fresher’s’ week with the aim of promoting their use across the campus catering outlets. This was followed by a ‘ditch the disposable’ campaign where a disposable coffee cup charge (‘latte levy’) was implemented across the campus [48]. Although there was an initial increase in the number of hot drink sales made in reusable cups, this plateaued at an average 20%–25% across all campus catering outlets. As previous efforts to eradicate single-use coffee cups across the campus had been of limited effectiveness, the university aims to develop of an intervention informed by behavioural science. The study is a collaboration between behavioural scientists at UCL’s Centre for Behaviour Change [49], the multi-disciplinary team at the Plastic Waste Innovation Hub [50], UCL’s Sustainability team [51], representatives from UCL’s catering team and Sheffield University’s plastics research and innovation hub [52].

## Discussion

Solving many of society’s sustainability challenges rely on changing human behaviour. A consideration of behaviour change is therefore critical for solutions aimed at sustaining environmental health. Seemingly simple behaviours, such as using single-use and reusable cups are located within complex systems of several interacting groups of actors (e.g., customers, manufacturers, suppliers, policy makers), operating across different groups (e.g., individual, community, population) and at various organisational levels (e.g., local, governmental). Behavioural science can aid in the designing of theory and evidence-based strategies that are more likely to be effective at achieving sustainable behaviour change.

There is a wealth of literature using behaviour change frameworks to understand, change and synthesise evidence related to health-significant behaviours [53–58]. However, applications of behaviour change science are required in many areas beyond this. Examples of TDF applied to understanding behaviours outside of healthcare include participation in citizen science [59], cybersecurity behaviour [60] and behavioural science evidence uptake [61]. Applications of COM-B outside of healthcare include understanding how to encourage higher welfare food choices [62] and data leakage in financial organisations [63].

There have been only a few published examples of COM-B and TDF applied to an environmentally-significant target behaviour. Such applications of TDF include a case study on understanding recycling at a London university [64]. Applications of COM-B include understanding purchase of biodegradable and compostable plastic packaging [47], plant-based diet adoption [65], household water conservation [66] and sustainable food choice [67]. The design of our method is therefore useful and novel in terms of its application within a sustainability context. We outline a clear sequence of activities for understanding single-use and reusable cup use and have illustrated its applicability within in a large metropolitan university context. It can serve as a template for understanding a wide variety of environmentally significant behaviours and foundation for designing interventions that sustain environmental health.

## Conclusion

Prior interventions aimed at changing citizens’ cup use have not been informed by behaviour change theory. The benefits of using integrative theoretical frameworks in behaviour change research include an improved understanding of the factors that encourage, hinder and/or maintain behaviour. When this evidence is applied to intervention development, this leads to the design of behaviour change strategies that are more likely to be effective. Our methodology provides an adaptable template, with guidance, that can be used by other intervention practitioners and researchers to design such theoretically informed interventions. By openly documenting our methods before carrying our studies we also increase the transparency of the behaviour change research process.

## Data Availability

The datasets generated during and/or analysed during the current study are available in the repository: https://osf.io/ujkwe/.
